# Mammographic Breast Density Patterns in Asymptomatic Mexican Women

**DOI:** 10.1155/2012/127485

**Published:** 2012-12-30

**Authors:** Ana Laura Calderón-Garcidueñas, Mónica Sanabria-Mondragón, Lourdes Hernández-Beltrán, Noé López-Amador, Ricardo M. Cerda-Flores

**Affiliations:** ^1^Instituto de Medicina Forense, Universidad Veracruzana, 94294 Boca del Río, VER, Mexico; ^2^Unidad de Medicina Familiar No. 2, Instituto Mexicano del Seguro Social, 43612 Tulancingo, HGO, Mexico; ^3^Centro Medico Nacional del Noreste, Instituto Mexicano del Seguro Social, UMAE 25, 64180 Monterrey, NL, Mexico; ^4^Facultad de Enfermeria, Universidad Autonoma de Nuevo Leon, 64460 Monterrey, NL, Mexico

## Abstract

Breast density (BD) is a risk factor for breast cancer. *Aims*. To describe BD patterns in asymptomatic Mexican women and the pathological mammographic findings. *Methods and Material*. Prospective, descriptive, and comparative study. Women answered a questionnaire and their mammograms were analyzed according to BI-RADS. Univariate (*χ* ^2^) and conditional logistic regression analyses were performed. *Results*. In 300 women studied the BD patterns were fat 56.7% (170), fibroglandular 29% (87), heterogeneously dense 5.7% (17), and dense pattern 8.6% (26). Prevalence of fat pattern was significantly different in women under 50 years (37.6%, 44/117) and older than 50 (68.8%, 126/183). Patterns of high breast density (BD) (dense + heterogeneously dense) were observed in 25.6% (30/117) of women ≤50 years and 7.1% (13/183) of women >50. Asymmetry in BD was observed in 22% (66/300). Compression cone ruled out underlying disease in 56 cases. In the remaining 10, biopsy revealed one fibroadenoma, one complex cyst, and 6 invasive and 2 intraductal carcinomas. 2.6% (8/300) of patients had non-palpable carcinomas. Benign lesions were observed in 63.3% (190/300) of cases, vascular calcification in 150 cases (78.9%), and fat necrosis in 38 cases (20%). *Conclusions*. Mexican women have a low percentage of high-density patterns.

## 1. Introduction

Breast cancer (BC) is a world health problem. Since 2006, it is the first neoplasm mortality cause in adult women in Mexico [1]. In 1990, 6000 new cases were registered and by 2020, 16,500 are expected. At present, most of these cases are detected by self-exam and only 10% are identified in stage I [1, 2]. An essential tool in early detection of BC is mammography. The advance of technology has refined the images with increased accuracy for detecting non-palpable lesions [3]. In Mexico, there is a limited number of mammography machines (4 per million population), below the OECD (Organisation for Economic Cooperation and Development) indicators (19.9 per million population) and far from developed countries (France, 42.2/million) [4].

Incidence of BC increases with age. In Mexico, 46% of BC cases occur before age 50 and the age group most affected is that between 40 and 49 years [5]. This contrasts with the United States, where the average age of presentation is 61 years [6] and with European countries where the incidence is higher in postmenopausal women [6–8]. Mexico's first voluntary mammography screening program was organized by the Mexican Foundation for Education in Prevention and Opportune Detection of Breast Cancer (FUCAM) and the Mexico City Government. It targeted women over 40. More than 96,000 mammograms were performed in mobile units for residents in Mexico City's Federal District over a 22-month period ending in December 2006. Out of 949 women with abnormal mammograms, 208 had breast cancer, a rate of 2.1%. Most were in situ, stage I (29.4%) or stage II (42.2%). One percent were in BI-RADS 0, 4, or 5. Of the women diagnosed with cancer, 68.5% were younger than 60, with an average age of 53.5 [9]. Thirty-eight percent of the cancers occurred in women aged 49 or younger [9]. 

The screening programs are regulated by the Mexican Official Standard (NOM-041-SSA2-2002) [10]. The NOM provides detection through self-examination, clinical examination, and mammography, the latter every one or two years in women 40 to 49 years with two or more risk factors, and annually for all women 50 years, whenever there is a resort [10]. 

Actually, the program currently covers 19% of the female population aged 40 or more [10]. 

Mammography is considered the most sensitive tool for detecting early neoplasia. A particular parameter analyzed in mammography is breast density. Mammographic density reflects variations in adipose, stromal, and epithelial tissues [11], associated with advancing age and hormonal changes experienced by the woman. Hence, it is an important parameter to evaluate and in fact, a risk factor for BC [12]. In the mammary gland, the older the woman, the higher fat content to be found. However, patterns at high risk of cancer have different distributions in different ages and populations. Breast density has the potential to be an important adjunct to risk estimation and to monitor interventions for breast cancer prevention with hormone replacement therapy and by change in life style behaviours [12].

 Therefore, the aim of this study is to determine the breast density (BD) patterns in a sample of asymptomatic Mexican women.

## 2. Material and Methods

The hospital is a referral center for cancer patients and is a training center for radiologists in mammography. The hospital supports some primary care clinics with mammograms (medical and educational purposes). Women from these clinics were recruited for the study. Upon approval by the Research and Ethics Committees, all consecutive women clinically asymptomatic in relation to breast disease who attended the Department of Radiology and Image (July 2009 to July 2010) as part of the program of Screening for Cancer were included in the study. The selection criteria were 40 years of age or older, clinically asymptomatic in relation to breast disease, sent for early detection of cancer, and who had not had previous malignant breast disease. Exclusion criteria included the refusal to sign the consent form, no time to answer the questionnaire, prior history of breast malignancy, or breast resection surgery.

 The patients signed informed consent, underwent mammography, and gave the required clinical information based on a questionnaire that was conducted in the visit for mammography. Mammography studies were analyzed according to the criteria of BI-RADS [13]. 

Because of the age difference in presentation of breast cancer in Mexico in relation to other countries, we evaluated two groups, women 50 years or younger and women older than 50 years (> and <50 years) [5].

**Figure 1:**
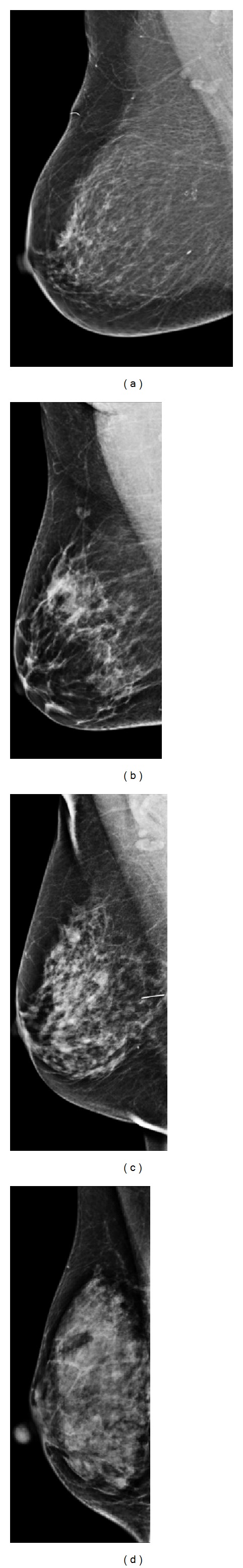
Breast density patterns according to percentage of glandular component: (a) fat (up 25%), (b) fibroglandular (25–50%), (c) heterogeneously dense (50–75%), and (d) dense (over 75%).

The BD was classified according to the percentage of glandular component (Figure 1): fat (up 25%), fibroglandular (25–50%), heterogeneously dense (50–75%), and dense (over 75%). For statistical evaluation purpose, fat and fibroglandular density patterns were considered “low density,” whereas heterogeneously dense and dense patterns were considered “high density.”

The technical quality of mammography was assessed at the time of the study [14] and mammary ultrasound (US) was performed if required. US was used as an adjunct to mammography, when the tissue was very dense, or because in the presence of a nodule, it was required to know whether the content was liquid or solid, also in cases of focal or global asymmetry, to rule out an underlying lesion not visible by mammography. Mammographic findings were assessed by two radiologists. In cases where classification and/or diagnosis were discordant, another radiologist evaluated independently the case and afterwards, the study was reviewed together to reach an agreement and a final consensus was obtained. Benign and malignant findings were described. We used a General Electric digital mammography, Senographe Model 2002, and two ultrasound machines Brand General Electric (model LOGIC 5, 2002, LOGIC 7.2003). The ultrasound machines were equipped with 7 MHz linear transducers and 3.5 MHz convex transducer.

19 variables were studied; 15 corresponded to the questionnaire: degree of obesity (according to Body Mass Index), age of menarche and menopause, tobacco (at least one cigarette per day) and alcohol consumption (at least one drink per week), contraceptive use (type and duration of use), hormone replacement therapy, number of pregnancies, cesarean sections and abortions, age and duration of the first lactation, history of mammography, who requested the study and family history of breast cancer, and 4 mammographic features (BD, BI-RADS and pathological findings, benign and malignant). Measurements of weight, height, waist, and hip were made in each patient. 

We performed univariate analysis (Chi-square tables) and multivariate (conditional logistic regression) using SPSS 17. Breast density was studied (1 high and 0 low) as well as its association with risk factors for breast cancer. The findings were compared in women younger and older than 50 years. 

## 3. Results

Of the 362 patients studied, 62 of them were excluded because they refused to sign the consent form (10/62, 16.1%), they had no time to answer the questionnaire (48/62, 77.4%), or there was a prior history of breast resection surgery (4/62, 6.4%). The study group was 300 women with age ranging from 40 to 77 years. Significant differences between the two groups were not observed in obesity degree (Tables 1 and 2), age of menarche (≤50: 12.68 ± 1.61; >50: 13.12 ± 1.64,  *t*-student −2.305, *P* = 0.022) tobacco and alcohol consumption, history of oral contraceptive use, history of pregnancies and abortions, lactation history, and familial history of BC (Table 2). No woman in the study was classified with abuse problem or alcohol dependency. Less than 20% of women were smoking, with an average of 5 cigarettes per day, ±2. 

 In these women aged 40 years or older, the present study was the first to be performed in 40% of cases. The average age of the first mammogram was 49.9 years, ranging from 40 to 71 years. The decision to seek mammography performed was the idea of the patient at 7% of cases and it was indicated by the physician or a nurse as part of the breast cancer screening program in 93%. There was no difference between the two groups in relation to family history of breast cancer in general, or in first-degree relatives (mother and sisters). As expected, menopause was present in less than 50% of women aged 50 years or younger and in 93% in older women. Women in the north of the country have the highest rate of caesarean sections in Mexico, a phenomenon that has already been detected since several years ago. 

Table 3 shows the distribution of BD patterns according to age. The general distribution of mammographic density in these women according to BI-RADS classification showed a fat pattern in 56.7% of cases, 29% with fibroglandular pattern, 5.7% with heterogeneous pattern, and 8.6% with high density pattern.

Table 4 shows BD and its relationship to women older and younger than 50 years. Mammographic density was strongly influenced by the age of the patient, with significant difference in younger and older than 50 years. Thus, the distribution of the four patterns, fat, fibroglandular, heterogeneous dense, and dense was 37.6, 36.7, 9.4, and 16.2%, respectively, for women under age 50 and 68.8, 24, 3.3 and 3.8% for women over 50 years. 

66 women (22%) showed asymmetry in mammographic density. The compression cone ruled out the underlying pathology in 56 cases. In the remaining 10 women, lesions were detected that were confirmed by biopsy. These lesions were diagnosed as fibroadenoma (1 case), complex cyst (1 case), and cancer (8 patients). Of the cancers found, 6 were invasive ductal carcinomas and 2 were intraductal neoplasms. All carcinomas were non-palpable lesions. Cancer was detected in 2.6% of patients in this study. 50% (*N* = 4) of cancers were found in patients 50 years or younger. 

Of the total sample, 190 women (63.3%) had benign lesions that corresponded to vascular calcifications (150 patients), fat necrosis (38 patients), and hamartomas (2 patients). 246 cases (82%) only needed the mammography for diagnosis, whereas in 54 patients (18%) it was necessary to supplement the study with ultrasound.

Mammography identified BI-RADS 1 in 78 patients (26%) of which only one required an additional ultrasonography (US) to define the diagnosis (0.3%); BI-RADS 2 was diagnosed in 190 patients (63.3%) of which the mammography was supplemented with US in 33 patients (11%). BI-RADS 3 was found in 16 patients, 2 hamartomas and 2 fibroadenomas, the remaining 12 patients needed to complement with US, detecting 5 simple cysts, 6 complex cysts and 1 cancer, BI-RADS 4 were 8 cases, and all required US complement; findings included 1 complex cyst, 1 fibroadenoma, and 6 neoplastic tumors.

Cancers detected by both mammography and US were later corroborated by biopsy. Of these, one was BIRAD 3, six had BI-RADS 4, and one was BI-RADS 5. Neoplastic cases were associated with fibroglandular density pattern in 3 cases, 3 had heterogeneous, and 2 high-density patterns. 5 carcinomas were found in 43 patients with high-density pattern (heterogeneous + dense) and 3 neoplasms were detected in 3/257 patients with low-density patterns (fat & fibroglandular) (Table 5). Table 5 shows the BI-RADS diagnosis according to BD patterns. Only dense density pattern in this sample was associated to BI-RADS 0. Also, it had the highest proportion of BI-RADS 3 and 4. The cancer cases were associated to high density in 5 cases (62.5%). 

## 4. Discussion

Breast cancer is a major cause of morbidity and mortality in our country. On the other hand, we have the growing problem of obesity [15]. Obesity is generally associated with an increased risk of developing breast cancer, which is most evident in women with morbid obesity [16]. Only 14% of the women studied were in normal weight and 17% were obese grades III and IV. Age and obesity in women in our population were correlated with low-density radiographic pattern (fat density, 56.7%). However, in spite of obesity 29% of these women had glandular pattern, 5.7% heterogeneous dense pattern, and 8.6% dense pattern. It is this latter group of patients in whom the risk of BC is higher. There was a significant difference in the patterns of breast density in women under age 50 as compared with older ones. The fat pattern increased from 38.5% in women under 50 years to 65.4% in the group of 56–60 years and reached 82% in women aged 61–65 years. However, even in old age it is possible to observe high BD, as the patients in the age group of 71–77 years. Breast high density is a risk factor for cancer. It is known that the incidence of breast cancer increases with age. In Mexico, 46% of BC cases occur before age 50 and the age group most affected is that between 40 and 49 years [5]. This contrasts with the United States, where the average age of presentation is 63 years [6, 8] and with European countries where the incidence is higher in postmenopausal women [7, 8]. Health authorities in Mexico recommend mammography from age 40 if there are at least two risk factors. 

When compared with other studies, the findings in Mexican women showed particular features. Mammographic density (percent dense area) was 60% or more in 8.3% of women in the UK [17]. In Korean women [18], the frequency of dense mammogram (heterogeneously dense and dense patterns) was 78.3% for women 40−44 years old, 61.1% for (45−49) group, and 30.1% in (50−54) age range. In Indian population a low prevalence of dense mammographic patterns (16.3% in noncancer controls and 26.7% in breast cancer cases) has been reported [19]. In Western women figures were 47.2% (40−44 years), 44.8% (45−49), and 44.4% (50−54) [19]. Mexican women had these frequencies of high density, 30.7% (40–45), 21.5% (46–50), and 3.7% (51–55). Therefore, the percentage of mammary glands with high density was lower than in other countries.

The presence of asymmetry in mammographic density requires to rule out underlying breast pathology. Besides additional projections, maximum compression, and lateral magnification cone to 90 degrees, the use of ultrasonography in our study was very useful to complement the characterization of lesions of the mammary gland [20]. US helped to define diagnosis specially in BI-RADS 3 and 4 in patients with high breast density pattern where the diagnosis included simple and complex cysts, fibroadenomas, and cancer. All patients with a final diagnosis of cancer by mammography and ultrasound underwent biopsy to corroborate diagnosis.

Coarse calcifications and fat necrosis were the most frequent benign lesions in this sample of Mexican women.

In this sample, 2.6% of patients had non-palpable cancer. The patients were referred for routine mammography. This finding is similar to the percentage found in the review of 96,000 mammograms in Mexico [9]. Carcinomas in this small sample were observed in 11.6% of women with high-density patterns, and in 1.1% of patients with low-density breast pattern, which supports the claim that high breast density is a risk factor for carcinoma.

## 5. Conclusion

 In general, Mexican women over 40 years of age have a breast density profile different from Asian and European women. 25.6% of women under 50 years of age and 7.1% of older women have high breast density. It would be interesting to compare the BD distribution patterns with other Latin American populations and determine if they are similar, especially due to risk of carcinoma in high breast density patterns. 

## Figures and Tables

**Table 1:** Body mass index (BMI) in 300 Mexican women.

BMI	Frequency	Percentage
Normal (18.5–24.9)	43	14.3%
Overweight (25–29.9)	96	32.0%
Grade 1 (30–34.9)	110	36.7%
Grade 2 (35–39.9)	37	12.3%
Grade 3 (≥40 kg/m^2^)	14	4.7%

Total	300	100%

**Table 2:** Distribution of characteristics according to age.

Characteristic	Age		*χ* ^2^	Probability *P*
	≤50 (117)	>50 (183)		
Normal weight	18 (15.4)	25 (13.7)		
Overweight-obesity	99 (84.6)	158 (86.3)	0.17	0.678
Menopause				
No	60 (51.3)	13 (7.1)		
Yes	57 (48.7)	170 (92.9)	75.65	0.000
Smoker				
No	98 (83.8)	151 (82.5)		
Yes	19 (16.2)	32 (17.5)	0.08	0.779
Alcohol consumption				
No	93 (79.5)	144 (78.7)		
Yes	24 (20.5)	39 (21.3)	0.03	0.868
History of oral contraceptives				
No	73 (62.4)	144 (66.7)		
Yes	24 (37.6)	39 (33.3)	0.57	0.449
Pregnancies				
No	10 (8.5)	12 (6.6)		
Yes	107 (91.5)	171 (93.4)	0.42	0.519
Cesarean section				
No	65 (55.6)	123 (67.2)		
Yes	52 (44.4)	60 (32.7)	4.15	0.042
Abortion				
No	90 (76.9)	122 (66.7)		
Yes	27 (23.1)	61 (33.3)	3.62	0.057
History of lactation				
No	21 (17.9)	27 (14.8)		
Yes	96 (82.1)	156 (85.2)	0.54	0.462
Previous mammography				
No	44 (37.6)	76 (41.5)		
Yes	73 (62.4)	107 (58.5)	0.46	0.499
Mammography required by:				
Physician	83 (70.9)	133 (72.7)		
Nurse (early detection program)	24 (20.5)	39 (21.3)	0.71	0.702
Women	10 (8.5)	11 (6.0)		
Breast cancer in the family				
No	86 (73.5)	150 (82.0)		
Yes	31 (26.5)	33 (18.0)	3.05	0.081
Mother with BC				
No	112 (95.7)	179 (97.8)		
Yes	5 (4.3)	4 (2.2)	1.07	0.301
Sister with BC				
No	107 (91.5)	175 (95.6)		
Yes	10 (8.5)	8 (4.4)	2.21	0.137

**Table 3:** Breast density, and age distribution.

Age (years)	Patterns				Total
	Fat	Fibroglandular	Heterogeneously dense	Dense	
40–45	19	17	7	9	52
46–50	25	26	4	10	65
51–55	53	25	0	3	81
56–60	35	11	3	3	52
61–65	23	4	1	0	28
66–70	11	4	1	0	16
71–77	4	0	1	1	6

Total	170 (56.7%)	87 (29%)	17 (5.7%)	26 (8.6%)	300

**Table 4:** Breast density in women younger and older than 50 years.

Mammographic density patterns	≤50 years		>50 years		Total
	*n*	%	*n*	%	
Fat	44	37.6	126	68.8	170
Fibroglandular	43	36.7	44	24.0	87
Heterogeneously dense	11	9.4	6	3.3	17
Dense	19	16.2	7	3.8	26

Total	117		183		300

*χ* ^2^ = 35.5, *P* = 0.0001, gl = 9.

**Table 5:** Distribution of mammographic diagnostics (BI-RADS) according to breast densities patterns.

BI-RADS	Density pattern				Total
	Fat	Fibroglandular	Heterogeneously dense	Dense	
BI-RADS 0	0	0	0	7 (26.9%)	7
BI-RADS 1	48 (28.2%)	29 (33.3%)	0	1 (3.8%)	78
BI-RADS 2	116 (68.2%)	51 (58.6%)	13 (76.5%)	10 (38.4%)	190
BI-RADS 3 # carcinomas	4 (2.4%)	5 (5.7%) 1*	1 (5.8%)	6 (23%)	16
BI-RADS 4 # carcinomas	2 (1.2%)	2 (2.3%) 2*	2 (11.7%) 2*	2 (7.6%) 2*	8
BI-RADS 5 # carcinomas			1 (5.8%) 1*		1

Total	170 (56.6%)	87 (29%)	17 (5.7%)	26 (8.7%)	300

*Number of carcinomas.
